# Afadin controls cell polarization and mitotic spindle orientation in developing cortical radial glia

**DOI:** 10.1186/s13064-017-0085-2

**Published:** 2017-05-08

**Authors:** Jennifer Rakotomamonjy, Molly Brunner, Christoph Jüschke, Keling Zang, Eric J. Huang, Louis F. Reichardt, Anjen Chenn

**Affiliations:** 1 Department of Pathology, University of Illinois at Chicago, 909 S Wolcott Avenue, Chicago, IL 60612 USA; 2 Department of Physiology, University of California, San Francisco, 1550 4th street, San Francisco, CA 94158-2611 USA; 3 Institute of Molecular Biotechnology of the Austrian Academy of Sciences (IMBA), Dr Bohr Gasse 3-5, 1030 Vienna, Austria; 4 Department of Pathology, University of California, San Francisco, 513 Parnassus Avenue, San Francisco, CA 94143-0502 USA

**Keywords:** Cortical development, Adherens junctions, Apicobasal polarity, Cell fate, Primary cilia

## Abstract

**Background:**

In developing tissues, cell polarity and tissue architecture play essential roles in the regulation of proliferation and differentiation. During cerebral cortical development, adherens junctions link highly polarized radial glial cells in a neurogenic niche that controls their behavior. How adherens junctions regulate radial glial cell polarity and/or differentiation in mammalian cortical development is poorly understood.

**Results:**

Conditional deletion of Afadin, a protein required for formation and maintenance of epithelial tissues, leads to abnormalities in radial glial cell polarity and subsequent loss of adherens junctions. We observed increased numbers of obliquely-oriented progenitor cell divisions, increased exit from the ventricular zone neuroepithelium, and increased production of intermediate progenitors.

**Conclusions:**

Together, these findings indicate that Afadin plays an essential role in regulating apical-basal polarity and adherens junction integrity of radial glial cells, and suggest that epithelial architecture plays an important role in radial glial identity by regulating mitotic orientation and preventing premature exit from the neurogenic niche.

**Electronic supplementary material:**

The online version of this article (doi:10.1186/s13064-017-0085-2) contains supplementary material, which is available to authorized users.

## Background

Cortical neurons develop from a layer of proliferating progenitor cells, called the ventricular zone (VZ), which lines the lateral ventricles of the embryonic brain. VZ progenitor cells, consisting of neuroepithelial cells and their progeny, radial glial cells, undergo symmetric cell divisions to expand the progenitor population or asymmetric divisions to produce more differentiated cell types, including more restricted progenitors and young neurons [1]. Additionally, as many aspects of cell fate are established at the time of a progenitor’s terminal division, the regulation of asymmetric and symmetric cell divisions plays a fundamental role in the generation of cell diversity in the developing cortex [2].

Recent studies have described unequal inheritance of determinants known to play important roles in establishing and maintaining asymmetric cell fate in mammalian cortical development [1]. Neuroepithelial and radial glial cells are highly polarized cells, having distinct apical and basolateral domains with characteristic morphology and associated protein complexes [3, 4]. This intrinsic apical-basal polarity provides an attractive mechanism to allocate subcellular determinants unequally during mitosis. By orienting the angle of mitotic cleavage, cleavage furrows could segregate determinants symmetrically or asymmetrically to produce equal or unequal daughter cells [5].

Although a number of apically-localized proteins have been described to regulate differentiation in the developing cortex through control of mitotic orientation [6–9], the factors controlling their polarized localization have not been identified. Neuroepithelial and radial glial cells are linked together by adherens junctions at the VZ lumenal surface [10]. Loss of adherens junction proteins in neural progenitors can lead to disrupted cell polarity, and alterations in proliferation [11–14] and differentiation [15, 16]. The roles for adherens junction proteins in cell polarity suggest that they may regulate proliferation and differentiation in neural development by controlling subcellular protein localization and asymmetric divisions. Relationships between apical-basal polarity, adherens junctions, and mitotic orientation remain poorly understood.

Afadin is an F-actin binding protein localized at cell-cell adhesion sites in epithelial cells and fibroblasts, and at adherens junctions in intestinal epithelial cells. Knockout mouse studies showed that Afadin is essential for cell adhesion, polarization, differentiation, and migration in the very early embryo [17]. More recently, Afadin was found to be highly expressed in VZ progenitor cells [18], and conditional deletion of Afadin from the cortical neuroepithelium resulted in loss of adherens junctions, increased proliferation, and abnormalities in radial glial morphology [12, 18]. Whether loss of Afadin led to changes in VZ cell polarity and mitotic orientation was not described.

Here, we investigated the role of Afadin in the in vivo regulation of the cortical progenitor pool by conditional deletion from cortical progenitors at embryonic day 9.5 (E9.5). We found that loss of Afadin led to altered radial glial cell polarity with mislocalization of Prominin-1 and primary cilia at E12.5. By E13.5, we observed widespread disruption of adherens junctions and redistribution of primary cilia away from the apical ventricular surface. Finally, we found increased numbers of mitotic divisions with oblique (non-planar) orientations, with mutant cortices characterized by increased generation of intermediate progenitors (IPs) and their progeny.

## Methods

### Mice

*Mllt4*-floxed mice have been previously described [19]. *Mllt4* ^*fl/fl*^ males were crossed with *Emx1-Cre*; *Mllt4* ^*fl/+*^ or *Emx1-Cre*; *Mllt4* ^*fl/fl*^ females. Mice were genotyped using standard PCR protocol.

### Tissue collection

Timed-pregnant females were euthanized and embryos were harvested. For adults, mice were anesthetized with Avertin 2.5% and intracardially perfused with successive solutions of PBS and paraformaldehyde (PFA) 4%. Brains were dissected and fixed with 4% PFA in PBS from 2 h to overnight. Brains were cryoprotected in sucrose 30%, embedded in OCT (Sakura Finetech) and frozen using a dry ice/ethanol bath and kept at −80 °C until use.

### Immunofluorescence

14-micron sections were cut on a cryostat (Leica) and stored at −80 °C until use. Sections were permeabilized with Triton 0.3% for 15 min. After a 15-min quenching step with 50 mM NH_4_Cl, sections were blocked either with 5% horse or donkey serum, 0.1% Triton X-100 in PBS, or with 5% fetal bovine serum, 1% BSA in PBS when the staining involved anti-Prominin-1 antibody. Sections were incubated overnight at 4 °C with primary antibodies in blocking solution, then with fluorescence-conjugated secondary antibodies, followed by DAPI or Sytox Green staining. Slices were mounted in Fluoromount-G (Clinisciences). Images were acquired using a LSM5 Pascal confocal microscope (Zeiss), a spectral C1si confocal microscope (Nikon), or a spinning disk confocal (Yokogawa CSU22) on a Nikon Ti-E inverted microscope.

### Spindle angle analysis

Coronal sections from E12.5 and E13.5 embryonic brains were stained with anti-α-tubulin, anti-γ-tubulin (Sigma-Aldrich), and anti-phospho-histone H3 (Abcam) antibodies as previously described [9]. Z-stacks images with a 0.5-micron z-step were taken using a spinning disk confocal. 3D-reconstruction was done with IMARIS software (Bitplane), and cleavage angle was determined as previously described [9, 20, 21].

### Electron microscopy

Embryo brains were fixed with 2% PFA/0.2% glutaraldehyde in 0.1 M phosphate buffer at pH 7.4 then cut into thick sections. Afadin was detected by the pre-embedding immunogold technique. After immunodetection, the sections were postfixed, dehydrated, and included in resin (Durcupan ACM; Fluka). Serial ultrathin sections were cut with a Reichert Ultracut S, contrasted with lead citrate, and analyzed using a Philips-FEI TECNAI 10 transmission electron microscope with SIA Micrograph Maxim DL5 software.

### Antibodies

Antibodies were as followed: N-cadherin (Reichardt Lab), pAb ZO-1, mAb Prominin-1, pAb PALS1 (Abcam), pAbs PAR3, Pax6, Tbr2 (Millipore), pAb Arl13b (Proteintech).

### Quantitative analysis

For progenitor quantifications, we counted Pax6+, Tbr2+, and double positive cells within a 100-μm-wide radial column. A minimum of 3 animals per genotype were used, with 2 sections per animal quantified. For quantifications from immunofluorescence and electron microscopy data, unpaired t-tests (Prism software, GraphPad) were used, to compare between control and *Mllt4*-KO mice, with significance level *p* < 0.05. Cell division angle quantifications were performed as described in [9] and Mann–Whitney test was used to compare between control and *Mllt4*-KO mice.

## Results

### Afadin is essential for the maintenance of adherens junctions in the neuroepithelium

**Fig. 1:**
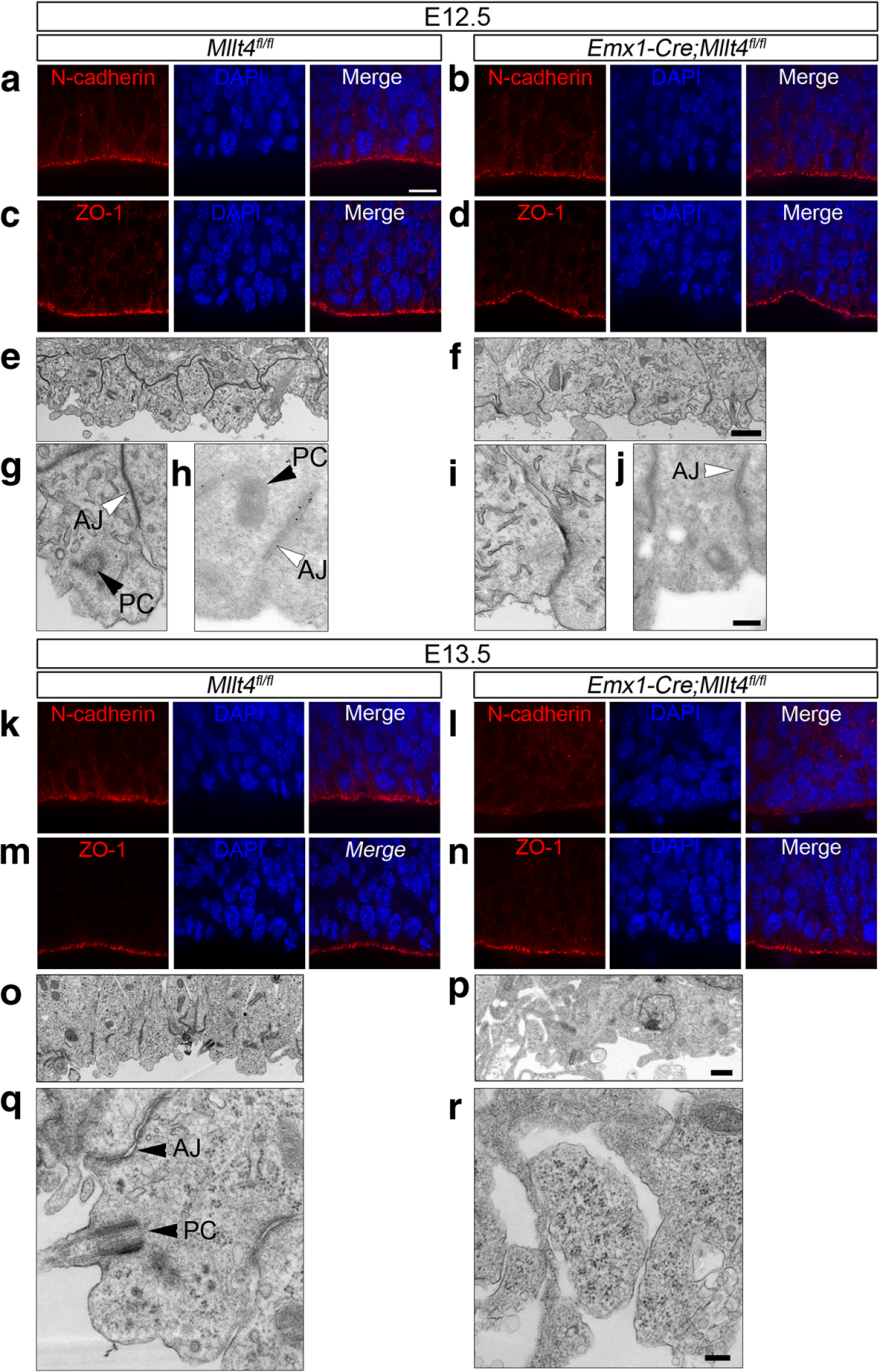
Afadin is crucial for maintenance of adherens junctions but not tight junctions in the dorsal telencephalon. At E12.5, in both (**a**) control (*Mllt4* ^*fl/fl*^) and (**b**) mutant (*Emx1-Cre*; *Mllt4* ^*fl/fl*^), N-cadherin is concentrated at adherens junctions. **c**, **d** The localization of the tight junction protein ZO-1 is similar in the control and mutant as well. **e-j** Electron micrographs of the E12.5 neuroepithelium at low (**e**, **f**) and high magnification (**g-j**) in control (**g**, **h**) and mutant (**i**, **j**) reveal that cells bordered with intact adherens junctions (*white arrowheads*) present a primary cilium basal body (*black arrowheads*). Mutant cells with disrupted adherens junctions (**i**) lack a primary cilium near the ventricular surface. Immunogold labeling of Afadin (**h** and **j**, right panels, *black dots* designated by *black arrows*) shows its expression at adherens junctions in control. (AJ, Adherens junctions; PC, Primary cilium). At E13.5, N-cadherin localization is disrupted in mutant (**l**) compared to control (**k**). **n** Expression of ZO-1 is not disrupted when compared to control (**m**). **o-r** Electron micrographs of E13.5 neuroepithelial cells at low (**o**, **p**) and high magnification (**q**, **r**) in control (**o**, **q**) and mutant (**p**, **r**) show that absence of Afadin further disorganizes cell-cell contacts with absence of a primary cilium at the ventricular surface. Scale bars: 10 μm (**a**-**d**; **k**-**n**); 1 μm (**e**, **f**, **o** and **p**); 0.2 μm (**g**-**j**, **q** and **r**)

Previous studies using Emx1-Cre (*Emx1-Cre; Mllt4* ^*fl/fl*^ mice) showed that conditional deletion of Afadin from developing cortical radial glial cells at E9.5 resulted in hyperproliferation and radial glial disorganization [12, 18]. To examine in detail the cellular and tissue architecture regulated by Afadin in cortical development, we first characterized the distribution of proteins found in adherens and tight junctions in cortical progenitors using the same *Emx1-Cre; Mllt4* ^*fl/fl*^ mice. We first confirmed that Afadin expression was effectively deleted at E12.5 (Additional file 1: Figure S1), making this stage our first time-point for our investigation. Despite the loss of Afadin at E12.5, immunofluorescence analyses did not reveal overt alterations in adherens junctions or tight junctions (Fig. 1a-d).

To examine whether the loss of Afadin impacted adherens junction ultrastructure beyond the resolution of light microscopy, we examined the radial glia apical endfeet using electron microscopy. In control animals, adherens junctions in the neural progenitors along the ventricular surface form compact electron dense structures, clearly defining the borders between cells (Fig. 1e, g, white arrowhead). In contrast, beginning at E12.5 in *Emx1-Cre; Mllt4* ^*fl/fl*^ mutants, some adherens junctions between neural progenitors appeared less compact with many long thin filamentous fibers (Fig. 1f, i). Immunogold staining using Afadin antibody confirms its expression along adherens junctions (Fig. 1h, arrows), whereas Afadin expression is substantially reduced in mutant adherens junctions (Fig. 1j).

At E13.5, we found evidence for additional adherens junction alterations, with N-cadherin immunoreactivity mislocalized from its normal distribution concentrated at the apical endfeet to the lateral membranes of radial glia in mutant mice (Fig. 1k, l). In contrast, the localization of the tight junction marker ZO-1 remained unchanged (Fig. 1m, n). Electron microscopy analyses at E13.5 confirm that adherens junction integrity at the ventricular surface is further disrupted in mutants (Fig. 1o-r).

Western-blot analysis on E13.5 dorsal cortex extracts to quantify AJ-component expression confirmed that Afadin expression level is substantially decreased in mutants. However, expression levels of N-cadherin, α- and β-catenin, p120ctn, pan-cadherin and ZO-1 are not significantly changed, arguing that mislocalization, not reduced expression of junctional proteins underlies the mutant phenotype (Additional file 2: Figure S2).

### Afadin is essential for the maintenance of cell polarity, primary cilia and anchoring of ciliated progenitors at the ventricular surface

**Fig. 2:**
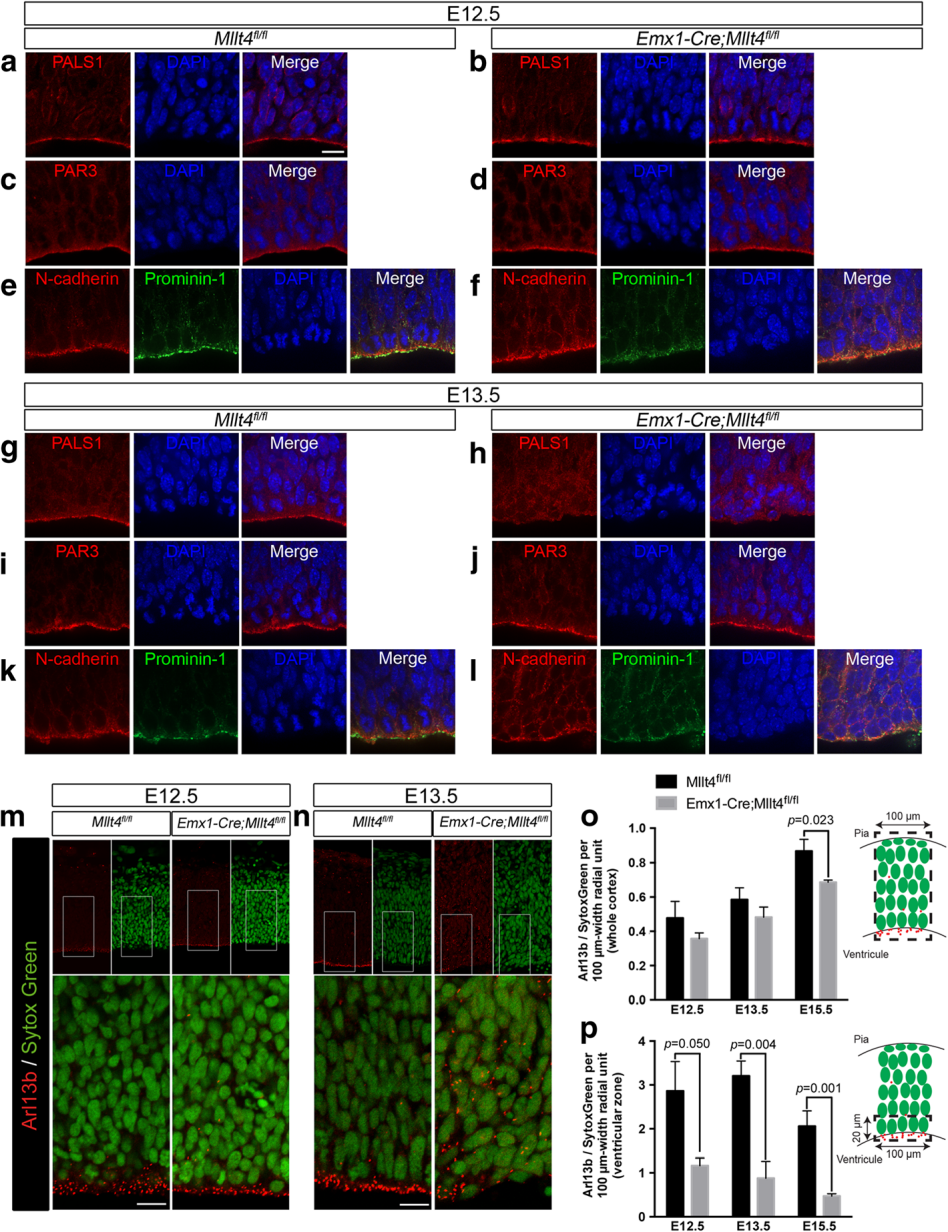
Apicobasal polarity loss and redistribution of primary cilia away from the ventricular surface in Afadin mutants. At E12.5, localization of the crumbs and Par complex-associated proteins (**a**, **b**) PALS1 and (**c**, **d**) PAR3 is similar in the control and mutant. **e** The apical domain protein Prominin-1 is localized adjacent to N-cadherin. In mutant, (**f**) localization of Prominin-1 is disrupted with elevated presence at lateral membranes of neuroepithelial cells lining the ventricle and in their cytoplasm. At E13.5, we observe disrupted localization of (**g**, **h**) PALS1 in the mutants, but not (**i**, **j**) PAR3. Prominin-1 localization, along with N-cadherin, is further disrupted in mutant (**l**) compared to control (**k**). **m**, **n** Lower (upper panels) and higher magnification images (lower panels) of the (**m**) E12.5 and (**n**) E13.5 dorsal telencephalon, visualizing the cilium-protein, Arl13b, in controls and mutants reveal the progressive loss of cilia from the ventricular surface in the mutant. (**o, p**) Quantifications of Arl13b puncta compared to Sytox *Green*-labeled nuclei (left panels) show that (**o**) total cilia number throughout the entire width of the telencephalon, as illustrated on the right panel, is not reduced in the mutant at E12.5 and E13.5, but decreases at E15.5. However, (**p**) the number of cilia within 20 μm of the ventricular surface, as illustrated on the right panel, is reduced at E12.5 and further decreased at E13.5 and E15.5 in mutant compared to control (mean ± s.e.m. Unpaired *t*-test; *n* = 3 embryos per group, 2 images per embryo). Scale bars: 10 μm (**a**-**l**); 20 μm (**m** and **n**, upper panels); 10 μm (**m** and **n**, lower panels)

In addition to adherens junctions, a variety of proteins localized apically at the ventricular surface (apical complex proteins) of radial glial cells play important roles in asymmetric cell division and regulation of cell fate in cortical development [22]. The known apical complexes are comprised of three inter-related complexes: Par3/Par6/aPKC, Crb/Pals1/Patj, and Mals/Pals1 [4]. To test whether Afadin regulates the expression or distribution of these apical complex proteins, we examined the distribution of PALS1 and PAR3. We found that both were unaltered at E12.5 despite the deletion of Afadin (Fig. 2a-d). At E13.5, however, the previously strong apical localization of PALS1 disappeared (Fig. 2g, h), while PAR3 localization remained intact (Fig. 2i, j), which we attribute to PAR3 involvement in tight junction assembly [23].

These findings that Afadin loss leads to alterations in adherens junctions and PALS1-containing apical complexes suggested that other apical proteins involved in cell fate might be abnormally localized. Prominin-1 is a cholesterol-binding membrane protein whose expression has been used to identify and purify stem cells from many cell populations [24]. Although the function of Prominin-1 in stem cell fate remains poorly understood, its asymmetric inheritance during cortical neurogenesis correlates with a potential role in stem-cell identity [25, 26]. Prominin-1 is distinctly localized apically at microvilli and primary cilium, and in the midbody of late-stage mitotic cells undergoing cytokinesis [27]. Surprisingly, prior to overt alterations in adherens junctions or other changes in cell polarity, we found that at E12.5, Prominin-1 was mislocalized to the lateral membrane and cytoplasm of radial glial cells in *Emx1-Cre; Mllt4* ^*fl/fl*^ mutants, in contrast to its restricted distribution along the ventricular surface, adjacent to N-cadherin in control embryos (Fig. 2e, f). Prominin-1 and N-cadherin were further mislocalized to basolateral membranes at E13.5 (Fig. 2k, l). Together, these results indicate that proper Prominin-1 subcellular localization requires Afadin.

Recent studies have provided evidence that the primary cilium plays important roles in normal brain patterning and development [22]. Prominin-1 is localized at the primary cilium in the neuroepithelium [28]. Although the presence of a primary cilium at the apical plasma membrane is a characteristic feature of radial glial progenitors [8, 29], little is known about their regulation in cortical development. Electron microscopy confirmed that neural progenitors in the cerebral cortex of E12.5 control embryos contain primary cilia localized at their apical endfeet (Fig. 1g, black arrowheads). However, primary cilia in the neural progenitors of *Emx1-Cre; Mllt4* ^*fl/fl*^ mutants are significantly reduced in number, specifically in cells bordered by altered adherens junctions (Fig. 1i). Quantification from electron micrographs shows that mutants had 63% fewer primary cilia at the ventricular surface than controls at E12.5 (Additional file 3: Figure S3A). This effect is specific to the area without Afadin (targeted by the Emx1-driven deletion) as the number of primary cilia in the ganglionic eminence is not significantly different between controls and mutants (Additional file 3: Figure S3A). Remarkably, by E13.5, we observed near total loss of apical cilia from the VZ in Afadin mutants (Fig. 1o-r).

To examine whether the reduction in VZ primary cilia in the developing dorsal forebrain results from disassembly, altered subcellular localization or cell displacement after Afadin deletion, we performed immunostaining for Arl13b, a small GTPase specifically localized to cilia, at different stages during the embryonic development. In control embryos, ciliated Arl13b-positive cells are present almost exclusively at the ventricular surface from E12.5 to E15.5. In contrast, in *Emx1-Cre*; *Mllt4* ^*fl/fl*^ embryos, Arl13b staining becomes increasingly re-distributed from the apical surface to basolateral positions throughout the cortical thickness (Fig. 2m, n). We detected no difference in the amount of Arl13b puncta/total cell number at E12.5 and E13.5 in the full thickness of the cortical wall (Fig. 2o), but at the ventricular surface, however, we observe 3.7- and 4.4-fold decreases for the same ratio at E13.5 and E15.5, respectively (Fig. 2p). Together, these results show that following Afadin deletion, the loss of primary cilia from the ventricular zone is a result of redistribution of ciliated cortical progenitors from their normal positions along the ventricular surface. Ciliated radial glial progenitors are anchored via adherens junctions at the neuroepithelium and their delamination is a characteristic of cerebral cortex development [8]. Thus, the massive dispersion of this cell population is consistent with premature delamination of neural progenitors from the ventricular neuroepithelium in Afadin mutants.

### Afadin deletion disrupts the distribution and proliferation of apical and intermediate progenitors

**Fig. 3:**
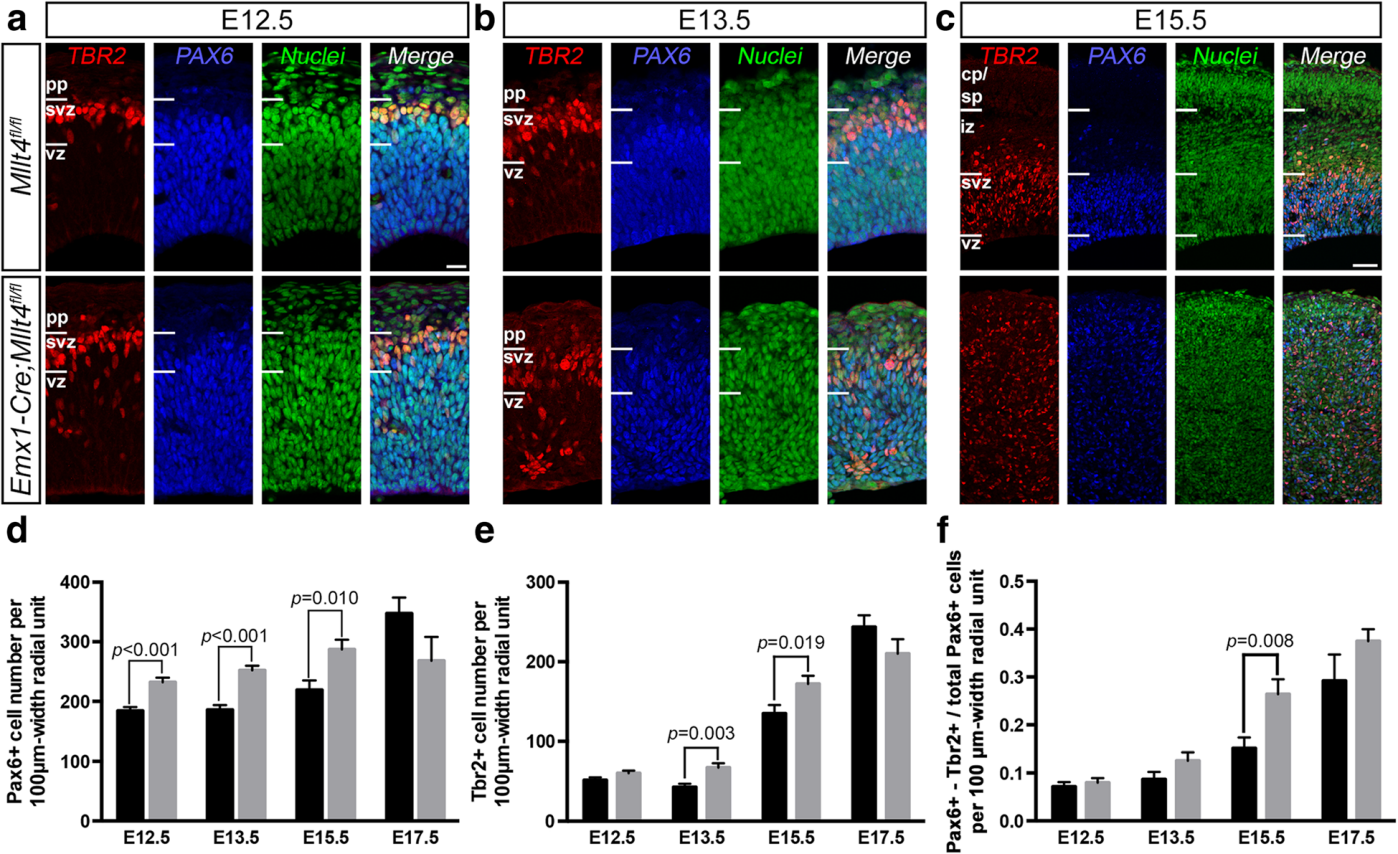
Afadin deletion leads to increased generation of apical and intermediate progenitors in the dorsal telencephalon. **a** - **c** Cortical layers are clearly defined in control brains (upper panels) including relative positions of apical progenitors (Pax6+) and intermediate progenitors (Tbr2+) in the ventricular and subventricular zones, respectively. Localization of these two precursor populations is increasingly disrupted in the mutant between E12.5 and E15.5 (lower panels) with widespread mislocalization of both cell types throughout most of the cortex in the mutant at E15.5. pp, preplate; svz, subventricular zone; vz, ventricular zone; iz, intermediate zone; sp, subplate; cp, cortical plate. **d**, **e** Statistical analyses of (**d**) apical and (**e**) intermediate progenitor numbers from E12.5 to E17.5 reveal that both progenitor populations see their number increase. **f** The fraction of Pax6+ cells double-labeled with Tbr2 increases as well at E15.5, indicating an enhanced transition from the apical to intermediate progenitor state following Afadin deletion (mean ± s.e.m. Unpaired *t*-test; *n* = 3 animals, 2 images per embryo). Scale bars: 20 μm (**a**, **b**); 50 μm (**c**)

The redistribution of the apical primary cilium to the basolateral membrane is the earliest known marker for cell commitment to delamination from the VZ as radial glial cells become IPs [8]. To examine other molecular markers of radial glia differentiation in the Afadin mutant cortex we assessed the expression of Pax6 and Tbr2, which label apical and intermediate progenitors in the developing cortex, respectively [30]. In control cortices, Pax6- and Tbr2-positive cells form distinctive layers (Fig. 3a - c, upper panels). In the *Emx1-Cre; Mllt4* ^*fl/fl*^ cortices, Pax6+ apical progenitors show significant increase as early as E12.5, and by E15.5 the localization of Pax6+ cells is diffuse and lacks defined boundaries (Fig. 3c, lower panels). Total cell number counts reveal a 26%, 36% and 31% increase in Pax6+ progenitor numbers at E12.5, E13.5 and E15.5, respectively (Fig. 3d). Total numbers of Tbr2+ IPs show a 57% and 28% increase at E13.5 and E15.5, respectively (Fig. 3e). Interestingly, overall fractions of Pax6+ cells double-labeled with Tbr2 are increased by 45% and 75% at E13.5 and E15.5 (Fig. 3f), supporting the idea of an increased transition from apical to intermediate progenitors in Afadin mutants. Together, these findings show that the normal laminar organization of Pax6 and Tbr2 expressing cells is disrupted in Afadin mutant cortices, with increased production of both Pax6 and Tbr2-expressing progenitors.

The intermediate progenitor population can undergo 1–3 more cell division cycles before neuronal differentiation [31, 32]. Thus, disruption of IP generation could greatly shift the neurogenic output [9]. We thus assessed the postmitotic neuron population in Afadin mutants and found a 82% and 68% increase in Tbr1+ postmitotic neurons at E15.5 and E17.5, respectively (not shown). Collectively, our results strongly suggest that Afadin is involved in the maintenance of the radial glial cell state.

### Afadin regulates mitotic spindle orientation in cortical progenitors

**Fig. 4:**
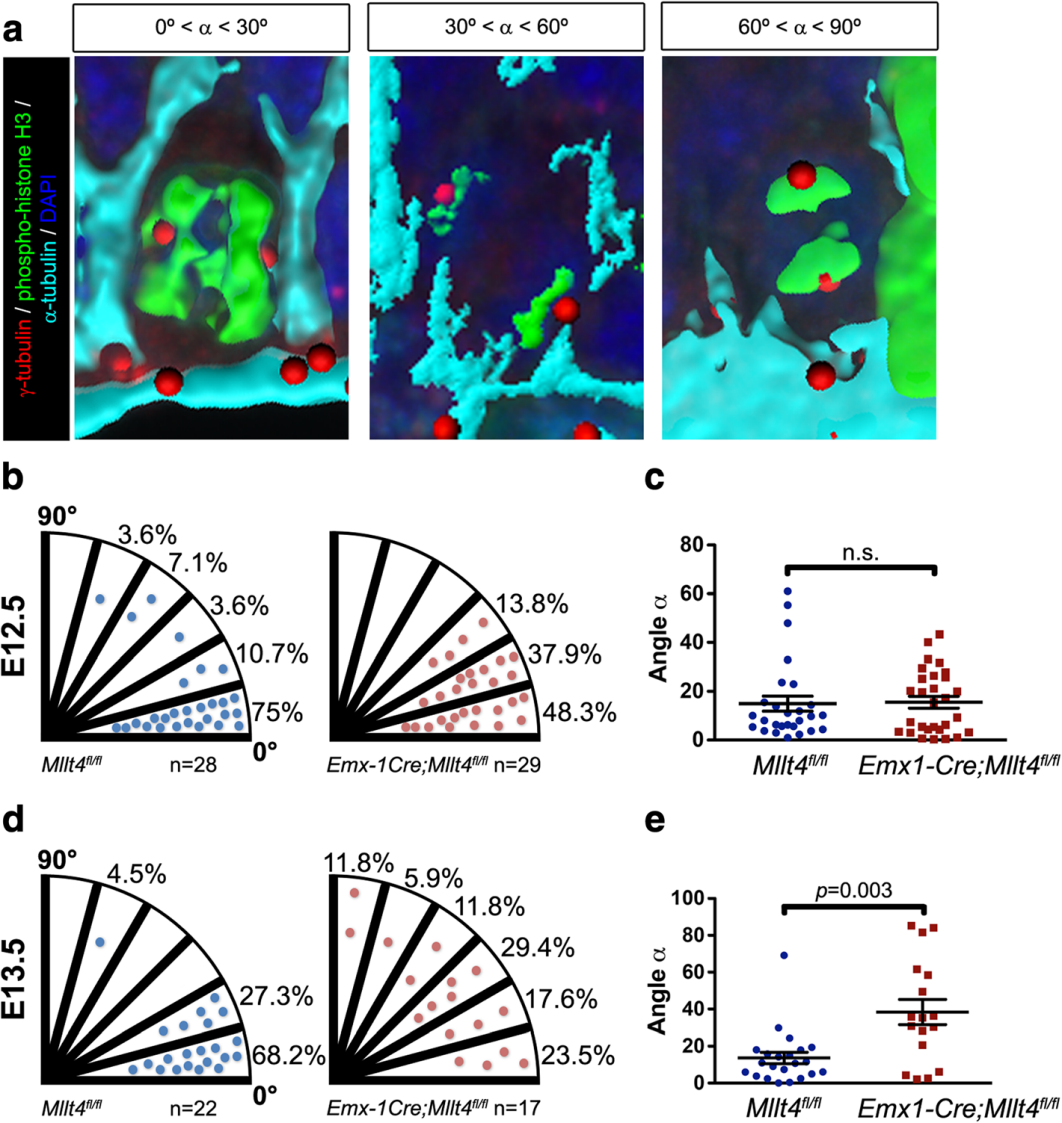
Afadin regulates mitotic spindle orientation in cortical progenitors at E13.5. **a** Samples of 3D-reconstruction of mitotic progenitors illustrating three different categories of mitotic spindle orientation in cell division. Cell borders are outlined with α-tubulin (*cyan*). ß-tubulin (*red*), phospho-histone H3 (*green*) and DAPI (*blue*) mark centrosomes, chromosomes and nuclei, respectively. **b** Bin distribution of individual spindle orientations in cortical progenitors at the ventricular surface of *Mllt4* ^*fl/fl*^ and *Emx1-Cre; Mllt4* ^*fl/fl*^ brains do not show significant difference at E12.5. **c** Distributions of mitotic spindle angles in control and mutant cells are similar at E12.5 (mean ± s.e.m. Mann–Whitney test). **d** Bin distribution of individual spindle orientations in cortical progenitors at E13.5 reveals that compared to controls, the mutant brain exhibits a much more random distribution of mitotic spindle division plane angles. **e** Distributions of mitotic spindle angles in control and mutant cells indicate that the average mitotic plane differs significantly between control and mutant at E13.5 (mean ± s.e.m. Mann–Whitney test)

During cortical neurogenesis, precise regulation of mitotic cleavage plane orientation has been found to play an important role in the control of symmetric and asymmetric division [33, 34]. Symmetric cell divisions have been proposed to segregate polarized apical determinants equally while asymmetric divisions result in unequal inheritance of these factors [35]. Abnormalities in mitotic cleavage orientation can lead to altered inheritance of cell fate determinants and changes in proliferation and differentiation [9, 36]. We thus hypothesized that Afadin-mediated regulation of the transition from apical to intermediate progenitors involves regulation of the mitotic cleavage orientation. We determined the orientation of mitotic spindles in control and *Emx1-Cre*; *Mllt4* ^*fl/fl*^ cortical progenitors in three dimensions at E12.5 and E13.5 (Fig. 4a) [9]. Control and mutant embryos at E12.5 both show predominantly vertical cell cleavage planes (angles α, control = 14.4° ± 3.0°; mutant = 15.3° ± 2.4°) (Fig. 4b, c). However, by E13.5, nearly 60% of progenitors in *Emx1-Cre; Mllt4* ^*fl/fl*^ mutants show an oblique or horizontal division plane (average angle α = 38.4° ± 6.8°) vs. only 4.5% in controls (average angle α = 13.6° ± 3.1°) (Fig. 4d, e). These findings indicate that Afadin is essential for maintaining the planar orientation of mitotic spindles.

## Discussion

**Fig. 5:**
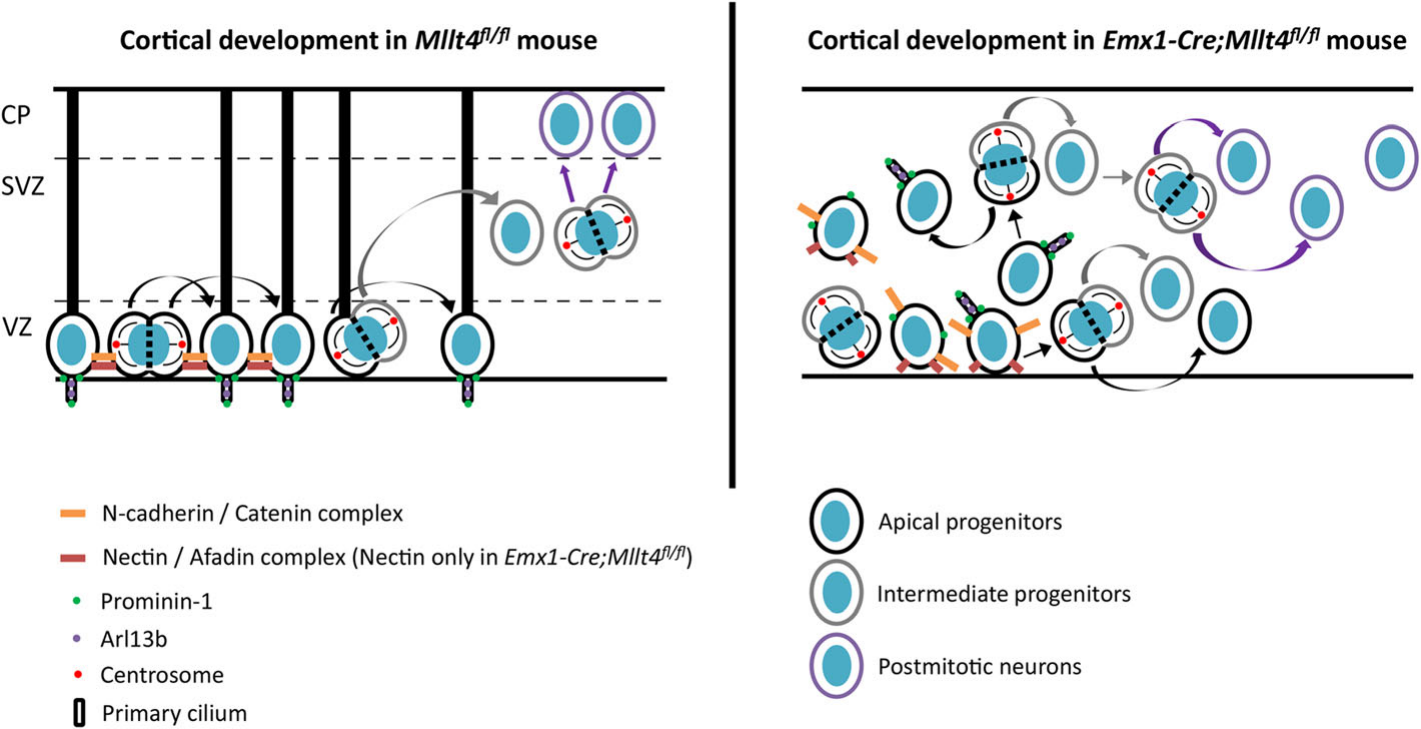
Shown is a schematic of cortical organization at E13.5 in control (left panel) and Afadin mutant (right panel). Afadin restricts apicobasal complexes and primary cilium at the apical membrane of radial glial progenitors. Cell division plans are mainly vertical, tangentially expanding the progenitor pool. In *Mllt4* knockout brains (right panel), random diffusion of AJ and apicobasal polarity-associated proteins is allowed, disrupting cell division orientation and triggering progenitor delamination, and premature fate determination. Disruption of the radial glial scaffold [18] allows random dispersion throughout the cortical wall VZ: ventricular zone; SVZ: subventricular zone; CP: cortical plate

Here we provide evidence that Afadin regulates apical-basal polarity, adherens junctions, and mitotic spindle orientation in radial glial cells in the developing cerebral cortex. Loss of AJs leads to randomized orientation of cell division, premature release and displacement from the ventricular neuroepithelium and premature neuronal differentiation of radial glia (Fig. 5).

The first abnormalities we detected in the developing cortical neuroepithelium of the Afadin mutant were altered subcellular localization of Prominin-1 and primary cilia at E12.5, followed by widespread loss of adherens junctions by E13.5. We found that Rab11, a small GTPase that functions in apical membrane trafficking, was also mislocalized in the Afadin mutant (Additional file 3: Figure S3B). Our findings support previous studies showing that localization and activation of Rab11 depends on Afadin, through phosphoinositide 3-kinase (PI3K)/Akt signaling [37, 38], and suggest that intact apical-basal intracellular trafficking is essential to maintain adherens junction structure.

In addition to the previously described roles of Afadin in cell proliferation and adherens junction maintenance in cortical development [12, 18], our findings provide new evidence that Afadin regulates mitotic division orientation of radial glial cells. The developing Afadin mutant cortices were characterized by increased numbers of obliquely-oriented cell divisions in the VZ. Consistent with observations that obliquely-oriented cell divisions preferentially generate intermediate cortical progenitors [9, 39], we also observed increased numbers of Tbr2 expressing progenitors in the Afadin mutant cortex.

The relationships between Afadin, epithelial cell polarity, adherens junctions, and mitotic spindle orientation are complex. The Drosophila counterpart of Afadin, Canoe is required for asymmetric division of neuroblasts oriented perpendicular to the epithelial plane [40]. In contrast, other studies suggested that polarity cues provided by adherens junctions could override apical-basal polarity cues to maintain cell division orientation within the epithelial plane and inhibit asymmetric division [41]. Supporting this role of adherens junctions in maintaining symmetric mitotic divisions, a recent study in human epithelial cells provided evidence that Afadin controls planar mitotic orientation within epithelia by binding F-actin and the protein LGN to direct spindle positioning [42]. Our observations that Afadin loss in the developing mammalian cortical neuroepithelium results in increased numbers of non-planar mitotic orientations provide further support for the role of Afadin and adherens junctions in inhibiting asymmetric division.

Previous studies have shown that Afadin’s deletion in the dorsal forebrain leads to an expanded population of Cux1 neurons mispositioned below the white matter, believed to result from increased proliferation [12] and migration defects caused by disruptions in the radial glial scaffold [18]. We analyzed the proliferative potential of cortical progenitors in our mice throughout embryonic development, from E11.5 to E17.5 (data not shown) and our results provide support for both studies. As Gil-Sanz et al. observed, we also found increased proliferation and decreased cell cycle exit in Afadin mutants, these effects being limited to an E12-E13 time-window. Consistent with the findings from Yamamoto et al., who examined E14.5 brains, we did not observe changes between E13.5 and E17.5. Minor increases in progenitor production can substantially expand progenitor populations [43] and provide for an early increase in apical and intermediate progenitor numbers observed in the Afadin mutant (Fig. 3) [12]. After this brief increase in progenitor proliferation, our findings showing that Afadin loss promotes premature production of IPs with an increased fraction of transitioning progenitors expressing both Pax6 and Tbr2 markers beginning at E13.5 (Fig. 3f), when Cux1 neurons are produced [44, 45]. These observations provide evidence that the surplus of Cux1 neurons in Afadin mutants arise from the expanded population of IPs and support previous observations that loss of IPs predominantly impact the number of upper layer neurons [46].

## Additional files

Additional file 1: Figure S1. Afadin expression in the developing dorsal forebrain. Immunofluorescence stainings using Afadin antibody show that Afadin is concentrated at the ventricular (apical) surface in the control at E11.5 (A) as well as E12.5 (C). Though still present at E11.5 in mutant (B), Afadin is absent from the neuroepithelium of the dorsal forebrain at E12.5 (D), consistent with the expression domain of Emx1-cre [47]. Scale bar: 10 μm (A – D). (TIF 8310 kb)

Additional file 2: Figure S2. Expression of adherens junction-associated proteins in control and Afadin-deleted dorsal forebrains at E13.5. (A, B) Western blot analysis of E13.5 dorsal forebrain extracts show a strong reduction of Afadin expression in mutant, but not of intercellular junctions-associated proteins α-catenin, N-cadherin, β-catenin, p120catenin, Z0-1, or any cadherin when compared to control (mean ± s.d. Unpaired *t*-test.; *n* = 3 to 8 animals). (TIF 2373 kb)

Additional file 3: Figure S3. Loss of primary cilia and mislocalization of primary ciliogenesis initiator Rab11 following Afadin deletion. (A) Quantification of primary cilium number from electronic microscopy analysis at E12.5 confirms the loss of primary cilia specifically at the dorsal neuroepithelium (mean ± s.e.m. Unpaired *t*-test; *n* = 4 controls, 20 to 24 images per embryo; *n* = 3 mutants 15 to 24 images per embryo). (B) Immunofluorescence for the small GTPase Rab11 in control (upper panels) and mutant (lower panels) reveals that Afadin allows the proper distribution of this initiator of primary ciliogenesis. (TIF 1961 kb)

## Abbreviations

aPKC: Atypical protein kinase C
BSA: Bovine serum albumin
CP: Cortical plate
DAPI: 4′, 6-Diamidino-2-Phenylindole
IP: Intermediate progenitor
IZ: Intermediate zone
PFA: Paraformaldehyde
PI3K: Phosphoinositide 3-kinase
PP: Preplate
RIPA: Radioimmunoprecipitation assay
SP: Subplate
SVZ: Subventricular zone
VZ: Ventricular zone
