# Supplementary figures and images for: Afadin controls cell polarization and mitotic spindle orientation in developing cortical radial glia
Source: Neural Dev. 2017 May 8;12:7. doi: 10.1186/s13064-017-0085-2 (PMC5422985; doi:10.1186/s13064-017-0085-2)

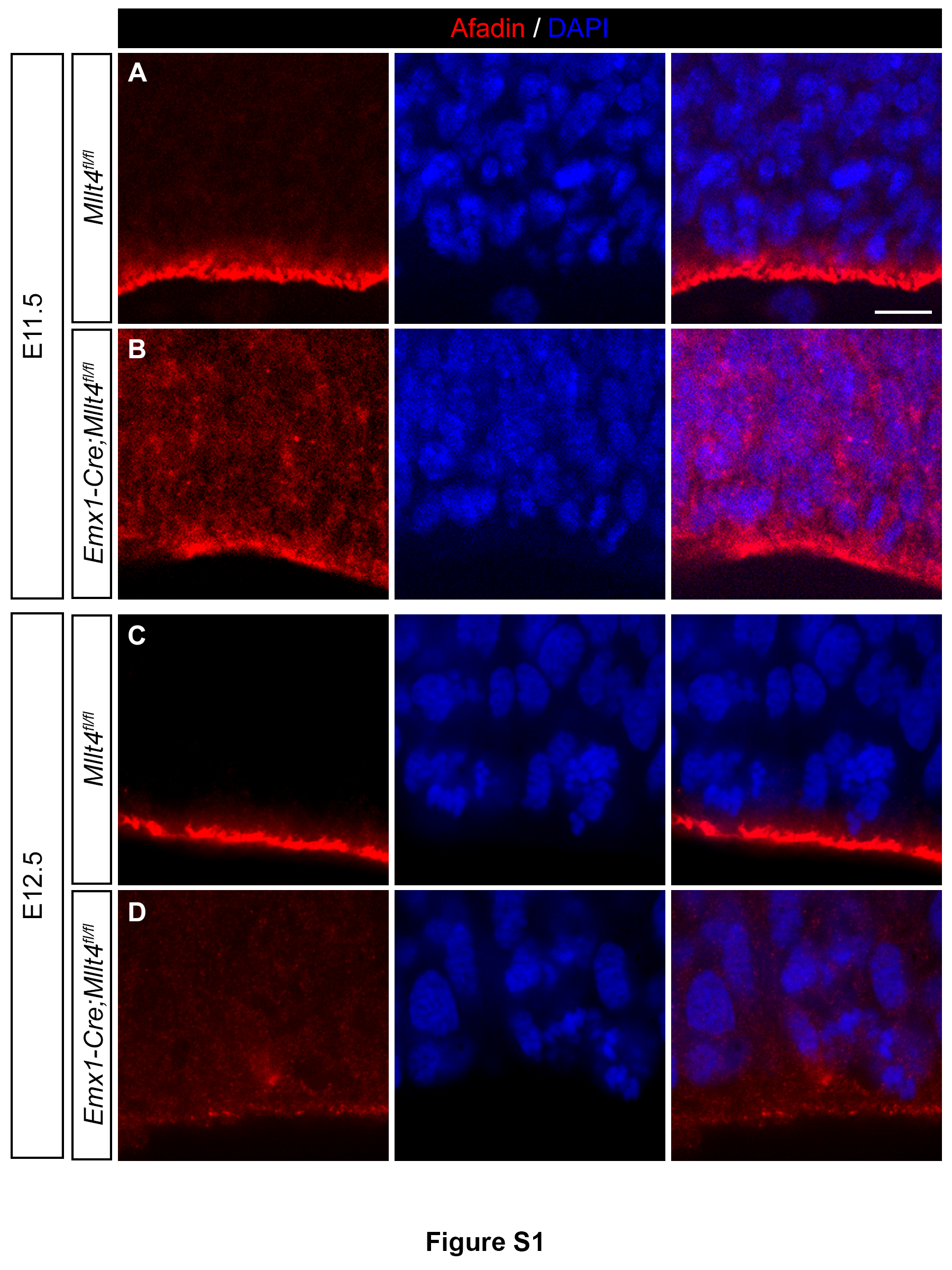

Supplement: Supplementary file 1 — Afadin expression in the developing dorsal forebrain. Immunofluorescence stainings using Afadin antibody show that Afadin is concentrated at the ventricular (apical) surface in the control at E11.5 (A) as well as E12.5 (C). Though still present at E11.5 in mutant (B), Afadin is absent from the neuroepithelium of the dorsal forebrain at E12.5 (D), consistent with the expression domain of Emx1-cre [47]. Scale bar: 10 μm (A – D). (TIF 8310 kb) [file 13064_2017_85_MOESM1_ESM.tif]

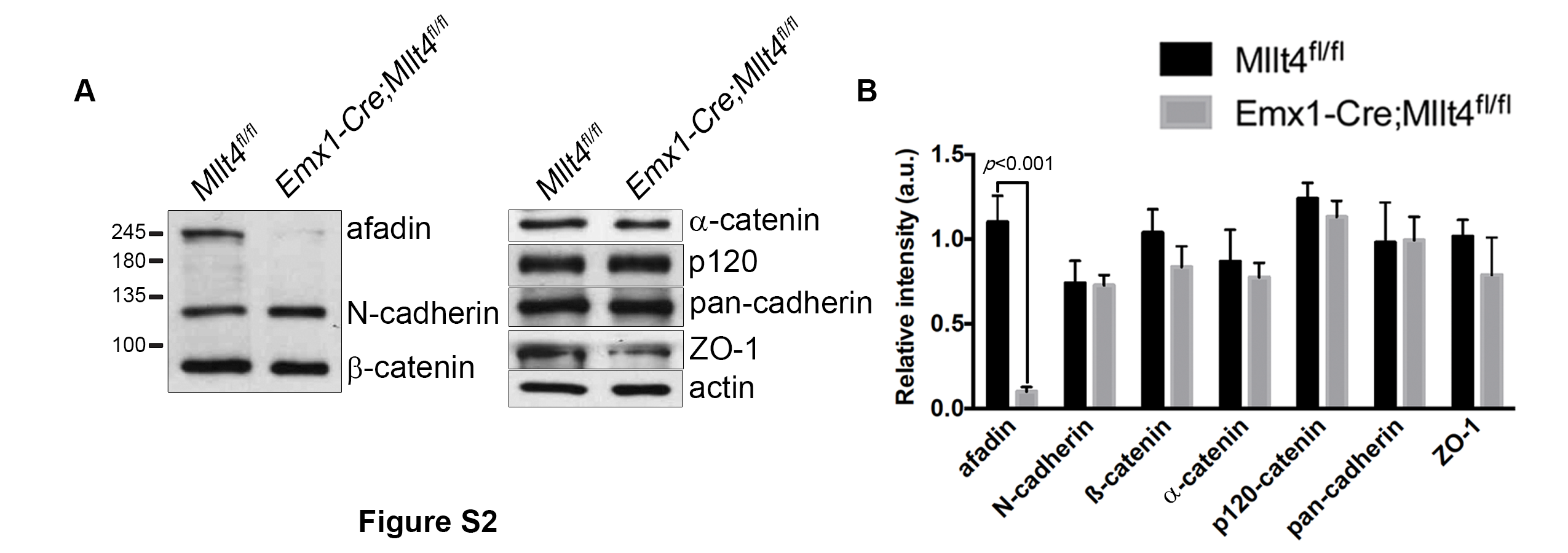

Supplement: Supplementary file 2 — Expression of adherens junction-associated proteins in control and Afadin-deleted dorsal forebrains at E13.5. (A, B) Western blot analysis of E13.5 dorsal forebrain extracts show a strong reduction of Afadin expression in mutant, but not of intercellular junctions-associated proteins α-catenin, N-cadherin, β-catenin, p120catenin, Z0-1, or any cadherin when compared to control (mean ± s.d. Unpaired t-test.; n = 3 to 8 animals). (TIF 2373 kb) [file 13064_2017_85_MOESM2_ESM.tif]

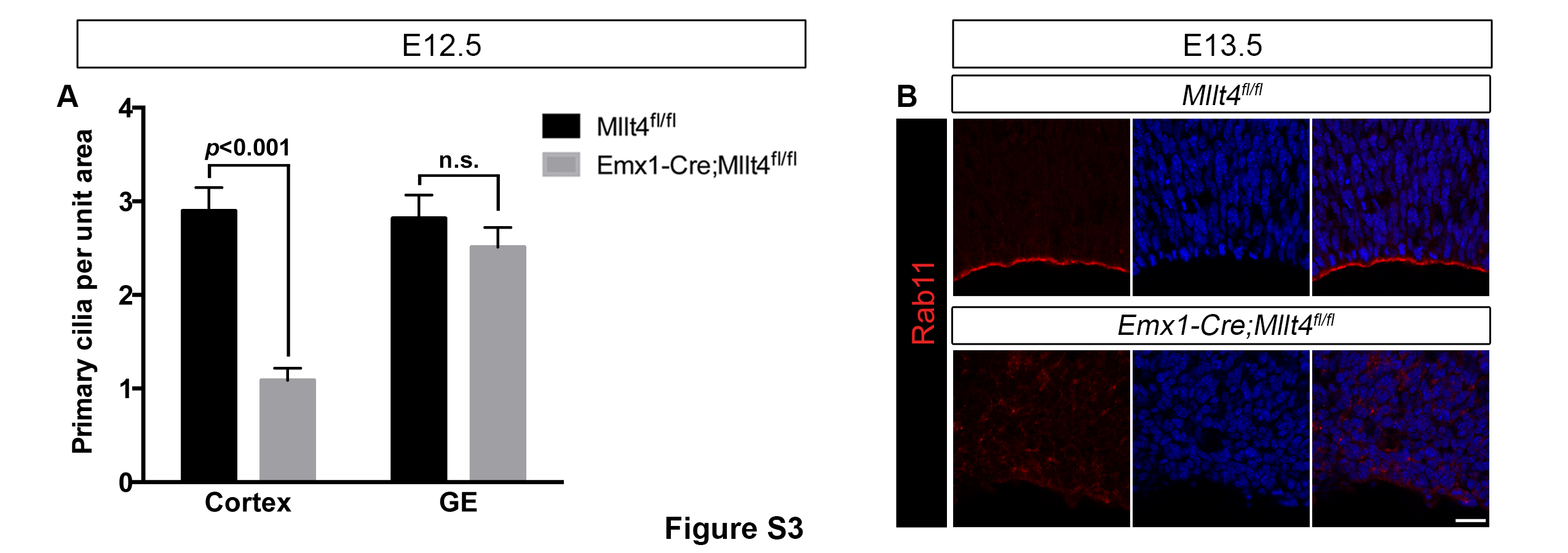

Supplement: Supplementary file 3 — Loss of primary cilia and mislocalization of primary ciliogenesis initiator Rab11 following Afadin deletion. (A) Quantification of primary cilium number from electronic microscopy analysis at E12.5 confirms the loss of primary cilia specifically at the dorsal neuroepithelium (mean ± s.e.m. Unpaired t-test; n = 4 controls, 20 to 24 images per embryo; n = 3 mutants 15 to 24 images per embryo). (B) Immunofluorescence for the small GTPase Rab11 in control (upper panels) and mutant (lower panels) reveals that Afadin allows the proper distribution of this initiator of primary ciliogenesis. (TIF 1961 kb) [file 13064_2017_85_MOESM3_ESM.tif]
